# Tracking the Current Rise of Chinese Pharmaceutical Bionanotechnology

**Published:** 2009-10-14

**Authors:** Tim Lenoir, Patrick Herron

**Affiliations:** 1 Jenkins Collaboratory, Duke University, 114 S. Buchanan Blvd., A103 Smith Warehouse, Box 90766, Durham, NC 27708

## Abstract

**ABSTRACT:**

**Background: The Context and Purpose of the Study**

Over the last decade China has emerged as a major producer of scientific publications, currently ranking second behind the US. During that time Chinese strategic policy initiatives have placed indigenous innovation at the heart of its economy while focusing internal R&D investments and the attraction of foreign investment in nanotechnology as one of their four top areas. China’s scientific research publication and nanotechnology research publication production has reached a rank of second in the world, behind only the US. Despite these impressive gains, some scholars argue that the quality of Chinese nanotech research is inferior to US research quality due to lower overall times cited rates, suggesting that the US is still the world leader. We combine citation analysis, text mining, mapping, and data visualization to gauge the development and application of nanotechnology in China, particularly in biopharmananotechnology, and to measure the impact of Chinese policy on nanotechnology research production.

**Results, the main findings**

Our text mining-based methods provide results that counter existing claims about Chinese nanotechnology research quality. Due in large part to its strategic innovation policy, China’s output of nanotechnology publications is on pace to surpass US production in or around 2012.A closer look at Chinese nanotechnology research literature reveals a large increase in research activity in China’s biopharmananotechnology research since the implementation in January, 2006 of China’s Medium & Long Term Scientific and Technological Development Plan Guidelines for the period 2006-2020 (“MLP”).

Since the implementation of the MLP, China has enjoyed a great deal of success producing bionano research findings while attracting a great deal of foreign investment from pharmaceutical corporations setting up advanced drug discovery operations. Given the combination of current scientific production growth as well as economic growth, a relatively low scientific capacity, and the ability of its policy to enhance such trends, China is in some sense already the new world leader in nanotechnology. Further, the Chinese national innovation system may be the new standard by which other national S&T policies should be measured.

## 1.1 Research Background

For more than two decades the Chinese government has been undertaking extensive reforms in its R&D system in order to create a modern national system of innovation (NIS). One defining characteristic of these reforms is the increasing importance of the role played by industrial enterprise in contrast to the controlling influence of Chinese government research institutes in the past.^1^ In nearly every category relevant to S&T development—in terms of percentage of Gross Domestic Product invested in R&D, growth in training of engineering, scientific, and R&D personnel, output of scientific and engineering publications, growth in patenting activity, development of R&D facilities and infrastructure, and attraction of foreign investment in R&D in China—the Chinese have made enormous strides. While much of the development was initiated during China’s ninth five-year plan (1996-2000), the most significant growth has occurred since 2000 under the tenth five-year plan (2001-2005) and since the launch of the new Medium- to-Long-Term Strategic Plan for the Development of Science and Technology (2006-20) (MLP). One goal of this paper is to examine the policy and strategic initiatives taken to build China’s current research infrastructure from 2000 through the beginnings and early years of the MLP. We discuss some of the policies aimed at stimulating indigenous innovation and their implementation in section 1.2 below. 

We are interested in the impact China’s strategic policy initiatives have had on nanotechnology, biotechnology, pharmaceutical research, and specifically on applications of nano and biotech in drug discovery, areas specifically targeted by the MLP as key areas for development. Tracking and assessing these developments poses special problems due to the dynamic, explosive, and rapidly changing development of the Chinese research and funding environment over the past decade. Studies relying on analysis of citations from core journals and patent applications in order to map the areas of nanotech have defined several subfields of activity, including nanobiotech, nanoelectronics, nanomaterials, nanodevices, nano-optics, and nanomagnetics.^2^ Such classifications provide at best a macro and static view of this dynamic and highly complex field. Moreover, as we discuss in more detail below, since such classifications are derived almost exclusively from citation patterns rather than directly from the internal content of the most recent publications, the maps have a built-in lag, potentially of several years. This problem is particularly troubling for our interest in pinpointing what is going on in the very near to recent past.

We are interested in constructing techniques for tracking the emergence of significant new areas of research in the fast-paced Chinese nanotech domain. We are also interested in assessing the quality of research in these emergent domains, identifying areas of intensifying interest, and assessing China’s success in meeting its policy goals in the area of biotechnology and pharmaceutical research. In section 2 of this paper we outline techniques of citation analysis, text mining, mapping and data visualization to focus on the intensifying development and application of bionanotechnology within the domain of pharmaceutical research in China. Our analyses of a comprehensive set of Chinese nanotechnology research articles identify recent globally significant innovation trends at the intersection of pharmaceutical technology and bionanotechnology taking place in China. These trends appear to support qualitative claims about the direction of Chinese bionanotechnology suggesting that China appears to be emerging as a superpower of scientific production, particularly in bionanotechnology.^3^,^4^

### 1.2 Stimulating Indigenous Innovation and the MLP

The stated aim of the Medium- to-Long-Term Strategic Plan for the Development of Science and Technology (2006-20) (MLP) announced in January 2006 is to transform China into an innovation oriented economy with strong indigenous innovation capacity. The most important aspects of the MLP can be summarized in three points: First, China will increase R&D expenditure as a share of GDP; Second, China will strengthen domestic innovative capacity and reduce dependence on foreign technology; Third, enterprises and the business sector will be the central driving force of the innovation process.^5^

To realize their objectives the framers of the MLP launched a multi-pronged strategy, including: 1) continued commitment to the training of highly qualified scientists, engineers, and research personnel who will provide the workforce for attracting offshore foreign research investment as well as the new domestic forces for fueling China’s indigenous innovation; 2) high investment in R&D facilities such as university labs and research parks; and 3) the protection of intellectual property rights of foreign firms in order to encourage foreign investment. While tremendous strides have already been made in transitioning from state owned enterprises to private industry in China, the MLP increases the focus upon improving infrastructure for financing R&D, funding innovative entrepreneurial activity, and creating incentives for encouraging small and medium-sized enterprises. 

The strategies for achieving the MLP’s goals continue program elements of the ninth (1996-2000) and tenth (2001-2005) five-year plans while increasing emphasis on the expansion of R&D personnel, strengthening of science and engineering education quality, and development of cutting-edge facilities. The MLP also supports a wide spectrum of framework conditions for indigenous innovation. The MLP calls for China to rank in the top five nations for granted invention patents, demanding that the quality of Chinese-authored papers rise to a ranking among the most highly cited papers in leading indicators such as the Science Citation Index (SCI), Engineering Index (EI), and the Index for Scientific and Technical Proceedings (ISTP). Science and Technology investment is a centerpiece of the MLP with investment of Gross Domestic Product in R&D increasing from the 1.42% GERD (ranking China third in percent of GDP investment in R&D behind the US and Japan) in 2006 to a projected 2.5% by 2020.^6^ The contributions to economic growth from technological advance, according to the MLP, will increase to more than 60% (double the current contribution), and dependence on imported technology will be limited to no more than 30% (currently dependence on imported technology exceeds 50%).^7^,^8^

Key to the MLP is a continued program of strengthening human resources for science and technology (HRST). This program includes the nurturing of scientific leaders and talent, tapping into the global HRST pool, encouraging the return of Chinese scientific leaders currently working abroad, reforming higher education, and improving public awareness of innovation. To meet these objectives China has taken a variety of measures to build indigenous human resources in science and technology (HRST) including increased investment in science and engineering education. The average annual growth between 2000 and 2005 was 18% (entrants), 23% (enrollments) and 26% (graduates). Owing to this recent growth, China has the world’s largest enrollments in higher education (Ministry of Science and Technology, 2006).^9^ The number of advanced degrees (master’s degrees and above) increased from 65,831 in 2000 to 189,728 in 2005, at an average annual growth rate of 24%. With 1.2 million scientist and engineer full-time equivalent researchers in 2006, China ranked second in human resources for R&D worldwide after the United States. Between 2001 and 2006 the total number of R&D personnel in China increased by 57% from around 950,000 to 1.5 million R&D personnel at an average annual growth of 5.9%, well exceeding the growth in R&D personnel by the US, Japan, and the EU nations. Measures have also been taken to assure that the quality of Chinese researchers is recognized as on a par with researchers of other leading S&T nations. For instance, Ph.D. students are now expected to publish at least one article in a journal listed in Thomson’s Science Citation Index; for more experienced academics, publication records are increasingly used to determine funding. Efforts to improve the quality of Chinese researchers has resulted in the Chinese science system being well connected internationally, as demonstrated by the number of Chinese publications with foreign co-authors, especially from the United States and Japan.^10^

China has taken measures to attract researchers to China as well as promote study abroad. The number of Chinese students studying abroad has increased steadily since the government reforms of the 1990s, and that trend has accelerated since 2000. In 2001, 84,000 students went abroad, while 118,515 Chinese students were studying abroad in 2005. In 2006 the Ministry of Education announced scholarships for study abroad in the fields of energy, life sciences, space, nanotechnology and new materials as well as several areas in the humanities and applied social sciences as priority areas for support. Important as background for developments in biotech and the pharmaceutical area is that the number of returnees has also steadily increased from a return rate of 15% in 2001 to more than 31% in 2006.^11^ Programs to attract high-level scientists, such as the “100 Talents” program of the Chinese Academy of Sciences and a similar initiative by the National Natural Science Foundation of China have aimed at encouraging Chinese researchers in other countries to return, offering attractive packages of competitive salaries, positions and research support.^12^ These returnees, nicknamed “sea turtles” by the Chinese (海龟,hǎiguī), have been viewed as a vital component of China’s innovation system, playing a key role in many of the country’s scientific and technological achievements, as well as its recent commercial successes. For example, in 2003, 45 incubators dedicated to returned overseas scholars hosted about 3000 enterprises employing more than 40,000 persons. According to a recent OECD study, Chinese returnees account for a high share of new businesses and knowledge production in terms of scientific publications, patenting and licensing. Many have been instrumental in setting up China-based R&D labs and institutes, both academic and corporate. Overseas returnees account for a significant portion of the foreign direct investment (FDI) flowing into China, and they are key personalities in China’s scientific community, including national chief scientists. This group has founded many of the country’s high-technology companies, and they have played a prominent role in prestigious scientific projects such as the space program and human genome mapping. In 2004, returnees accounted for 81% of the academicians of the Chinese Academy of Sciences (People’s Daily, 2 March 2004). More recently, they constitute a vital resource pool for foreign companies seeking to recruit at management level in China (China Daily, 13 March 2006). Overall, returnees provide unique access to networks, skills and funding sources.^13^,^14^

The quantity and quality of human resources in China have risen significantly in recent years. China’s increasing research strength, combined with its well-equipped laboratories and large supply of relatively inexpensive scientists and engineers—research personnel costs are roughly 30% of costs in the US or EU countries—attracts both the attention and the investments of many R&D-intensive companies. The quality of its researchers and research facilities have contributed to one of the most noteworthy changes in the Chinese NIS landscape; namely, the rapid increase in the number of R&D centers established by foreign companies. According to Western researchers there were 199 foreign R&D facilities in China in the beginning of 2004.^15^ Schwaag Serger argues that the number has since increased rapidly, possibly to around 350-450 foreign R&D centers by early 2007.^16^ Foreign investment in R&D is expanding rapidly and the reasons why foreign firms are investing in China are changing. The time when active foreign investors invested in China only to take advantage of cheap manufacturing platforms is over. Access to human resources has become a more significant factor. For instance, as we discuss below, recognizing the availability of high-quality researchers and state of the art infrastructure for research facilities pharmaceutical firms such as GlaxoSmithKline, Sanofi-Aventis, and others have opened their own dedicated research facilities in China as well as collaborative operations with several key science centers; and companies such as Intel and Microsoft have recognized Chinese-educated and trained scientists, engineers, and managers “are capable of doing any engineering job, any software job, and managerial job that people in the US are capable of doing.”^17^

A key, if controversial, cornerstone of the MLP is aggressive continuation of the policy of the previous five-year plans of encouraging direct foreign investment by high-technology firms through the establishment of foreign wholly-owned research facilities, joint ventures with Chinese firms, and collaborative arrangements with Chinese university and private research institutes. On the Chinese side, these arrangements offer the possibility of access to advanced technological and scientific knowledge and skills, opportunities to engage in advanced research, enhancement of management skills, access to up-to-date R&D equipment and, more generally, the improvement of research capacity. Over the course of the ninth and tenth five-year plans, the Chinese government actively promoted foreign corporate R&D in China, viewing it as a way to upgrade domestic technology and skills by importing, and ideally internalizing, foreign know-how. It remains unclear whether China can perform the balancing act of encouraging foreign investment in high-technology while successfully absorbing spillovers from foreign investment in order to stimulate its own indigenous innovation.^18^ In essence what is required is a triad of investment strategies of multi-national corporations engaged in foreign direct investment, a national government that constructs indigenous science and technology infrastructures, and indigenous companies that build on the investment strategies of foreign companies and the domestic government to become world-class competitors in their own right. As we explain in detail below, so far the results have been mixed at best. 

An essential first step in encouraging foreign direct investment and joint ventures was China’s joining the World Trade Organization in 2001 and reorganizing its intellectual property regime through acceptance of the TRIPS (Trade Related Aspects of Intellectual Property Rights) agreement. Article 27.1 (3) of TRIPS requires product patent protection for pharmaceutical products. The Chinese government also revised its patent legislation to extend the patent protection period from 15 to 20 years. Enhanced IP protection has been aimed at strengthening innovative activities of Chinese firms and also helping multi-national corporations to expedite their R&D outsourcing to China in areas such as drug discovery; these new protections are widely acknowledged within the pharmaceutical industry as effective enough to have been a crucial factor in the increase of foreign pharmaceutical R&D investment. 

These policies have had a significant impact. After 2001, when China joined the World Trade Organization (WTO), the amount of FDI increased rapidly. As of 2009 about one-third of China’s GDP and 57.44% of all exports and imports are due to foreign-invested firms. Even more striking in the context of the present discussion is that roughly 88% of all high-tech exports are due to foreign-invested firms and joint ventures.^19^,^20^,^21^ Thus, while the program to open its doors and encourage foreign investment has been extremely successful, some researchers are left wondering how much of China’s high tech export is actually Chinese^22^; and some question whether the strategy of absorbing spillovers of foreign investment in R&D is effectively stimulating indigenous innovation in China.^23^ The reason for concern is evident when we consider that in 2005 and 2006 only 38% and 43% respectively of patents granted by the State Intellectual Property Office (SIPO) in China were invention patents filed by domestic entities, whereas foreign invention patents granted in China comprised 62% and 57% of the totals respectively in those years. Such data prompted Cheng Siwei, Deputy Chairman of the Standing Committee of the National People's Congress to comment at the May 24, 2006 opening of the International Forum on the Development of China's High-tech Enterprises of the 9th China Beijing International High-Tech Expo that science and technology only contribute 30% of China's economic development and that China imports more than 50% of its technology. Perhaps projecting a note of irony, Cheng stated that building indigenous innovation is a slow process.^24^

However there are signs those efforts to build structures supportive of innovation are beginning to take root. One promising sign is that while invention patents are low in the overall composition of domestic patent applications to the Chinese State Intellectual Property Office (SIPO), their numbers have been growing at a very substantial pace—a 33% average annual increase from 2001-2006.^25^ At the same time China’s performance in world rankings of the World Intellectual Property Organization (WIPO) in terms of patents filed under the Patent Co-operation Treaty (PCT) in the three major patent offices—Japan Patent Office, US Patent & Trademark Office and European Patent Office—has skyrocketed. Completely unranked prior to 2000, between 2000-2005 China’s PCT patent filings increased at an average annual growth rate of 33% and moved progressively from tenth most filings in 2005 (with a 44% increase over 2004), to seventh place in 2006 (with a 57% increase over the previous year, ranking China number one in terms of filing growth rate).^26^ China ranked number 7 in filings to the PCT in 2007 and number 6 in 2008. Also in 2008 for the first time a Chinese company—Huawei Technologies, a telecommunications firm—ranked number one on the list of PCT applicant filings with 1,737 filings.^27^ Although the results for 2009 are provisional, as of April, 2009, China ranks number 4 in terms of total number of PCT filings.^28^

The goal of the MLP is not just to stimulate foreign investment; the aim is to leverage foreign technology investment to make indigenous innovation in China leading-edge. In addition to enhanced intellectual property rights (IPR) protection, key features of the MLP aimed at stimulating indigenous innovation and encouraging foreign investment are active participation in setting international technology standards, and further R&D infrastructure construction, including construction of key labs, science parks and incubators. The MLP calls for the adoption of Western laboratory standards and other related standards such as Good Supply Practices (GSP), Good Manufacturing Practices standards, and Good Use Practices (GUP); and in its 11th Five-Year Plan (2006-2010), China underlined its commitment toward drug development and manufacturing through the New Drug R&D Coordinating and Leading Group. China’s adoption of stringent domestic technical requirements and standards in areas of information technology and communications has been a significant factor in drawing companies such as Motorola, Microsoft, Ericsson, Sony Ericsson and Nokia as among the first to set up extensive R&D operations in China.^29^ China’s policy is to develop national standards in several high-technology fields, particularly information technology (IT), telecommunications, and biotechnology. As Suttmeier and Yao note, this policy is driven both by an ambition to promote the development of internationally successful Chinese high-technology firms and also by a desire to appropriate a greater share of the gains from globalization and innovation.^30^

A critical area for implementation of the MLP has been further investment in university science parks and incubators, high-technology industry zones, preferential tax incentives, and a diverse array of financial support for small-medium sized enterprises (less than 300 employees). University science parks are considered a base for technological innovation by strengthening university-industry linkages, nurturing high-technology start-ups, training innovative talent and diffusing technology. In 2001, the Ministry of Science & Technology (MOST) certified 22 national-level university science parks, and another 21 were created in 2002. In 2008 there were 49 university science parks with 49 incubators.^31^ In these incubators and science parks the MLP is using fiscal policy to stimulate innovation and the formation of technology-based startups. One of the most novel policies is the provision of tax incentives designed to encourage company R&D investments. For instance, one highly attractive incentive makes new R&D expenditure 150% tax deductible, effectively constituting a net subsidy, as well as introducing accelerated depreciation for R&D equipment worth up to 300,000 RMB.^32^ Another policy aimed at promoting indigenous innovation is the public procurement of technology, a strategy used by OECD countries. In the past the Chinese government has used public procurement to cut costs rather than promote indigenous innovation, but the new policy of the MLP on public procurement gives priority to indigenous innovative products in public procurement in terms of price and volume.^33^

China’s large investment in the development of science parks and incubators has paid off in the rapid development of small technology-based firms. In 2004, as much as 20% of the R&D personnel employed in domestic firms (excluding joint ventures with foreign partners) were in small enterprises (fewer than 300 employees). The OECD reports that between 2000 and 2004, the number of small S&T firms increased by 52%, the value added by these firms to economic output grew by 141%, and numbers of invention patents filed by these firms grew by 221%.^34^ A recent study by Lundin, et al. suggests that while small firms account for a relatively small share (9%) of total S&T development in China and most small firms are not engaged in any S&T, those small firms that do engage in S&T development tend to be more S&T intensive and have a higher output in terms of patents than larger Chinese S&T firms.^35^

These strategies have been highly successful in building an infrastructure to stimulate innovation in major areas of science and technology, including information and communications technologies (ICT), nanotechnology, and biopharma. China’s dedication to R&D investment has paid off in the production of scientific research and publication. The Chinese share of total world scientific article production increased from 2% in 1996 to 4% in 2004.^36^ China’s average annual growth rate in production of scientific papers was 14% over this period with the most dramatic increases in growth coming in the most recent years, indeed a growth rate of 17.6% from 2003- 2004.^37^ The Chinese only entered the top ten world producers of scientific articles in 1998. A decade later in 2008 China ranked second in the world behind only the USA, leapfrogging the UK, Japan and Germany in short order, a trend recognized by numerous researchers.^38^,^39^,^40^

Like other worldwide leaders in the funding of research and development, China has anticipated that nanotechnology will provide a broad base of new devices and materials with potentially unprecedented impact on many areas of the global economy. China clearly intends to be a major player in the nanotech arena. China was one of the world leaders in investment in nanotechnology before it had declared nanotech R&D as one of the four major foci of the MLP in 2006.^41^ A 2005 study by the European Commission on Nanosciences and Nanotechnologies listed China as ninth in absolute funding for nanotechnology research in 2004 and noted that if purchasing power were taken into account China would rank third on the list behind the US and Japan.^42^,^43^ China’s investment in developing infrastructure to support nanotechnology has resulted in dramatic increases in production of nanotechnology scientific articles as well: China ranked second in the production of publications related to nanoscience and nanotechnology as of 2007.^44^

The strategy of recruiting foreign investment while simultaneously improving the full spectrum of support structures for HRST has certainly paid off in building indigenous innovation in information and communications technologies (ICT). Lu and Lazonic have documented how the leading Chinese computer companies, Stone, Legend, Founder, China Great Wall Computing Company (CGC), and Lenovo partnered with Chinese state agencies, university labs, incubators and foreign companies much more powerful than themselves to gain access to technology and how they leveraged critical investments in manufacturing, R&D and marketing to move up the value chain from assembling and manufacturing computer boards to designing, manufacturing and distributing their own systems.^45^,^46^ The MLP states as one of its major benchmarks that China should move into the top five in the filing of invention patents. From 1995 to 2005 as the strategic plans outlined above were implemented China moved from being ranked twenty-second in ICT filings with the PCT to being ranked fifteenth in ICT filings in 2000 with 0.56% of the world’s total ICT patents, and moved into fifth place (behind the US, Japan, Germany, and Korea) in 2005 with 4.2% of the world’s total. The cumulative average growth rate for China’s ICT filings over the period 1995-2000 was a staggering 63%, by far and away the most rapidly developing country in this arena.^47^

It seems paradoxical that while life science institutes and biotechnology companies in North America and Europe have an ever-increasing percentage of Chinese-born scientists and entrepreneurs providing brains and muscle in the laboratory and in the boardroom Chinese firms and research institutes are not regarded as on the cutting edge of innovation. While China had developed considerable capacity in biopharmaceutical manufacturing prior to 2000, its strengths were in reverse engineering the products of brand name pharmaceuticals and producing generics. To move up the value chain the Chinese government recognizes it must develop more of its own indigenous R&D capabilities in pharmaceuticals while acting more aggressively to stimulate patenting of inventions. The policies that have guided the development of China’s national innovation system have succeeded in creating an attractive environment for foreign investment and in luring foreign firms to locate research and development facilities in China. The question is whether the Chinese will be able to take advantage of these developments in ways that advance their own indigenous science and research capabilities in areas of nano- and biotechnology. We believe they are on the verge of doing just that, and they are using their successful strategy in building ICT as a blueprint for building indigenous capacity in biopharmanano.

In this section we have discussed policy measures implemented by the MLP targeted at growing the research economy and stimulating indigenous innovation. Among the fields targeted for growth under the MLP are biotech, nanotechnology, and biopharma. In order to assess the effectiveness of the MLP we consider the growth of Chinese contributions to nanotechnology in the very recent past. Nanotech covers a wide range of fields; so we are interested in distinguishing among different fields of work within the growing population of nanotechnology documents related especially to bio/nano/pharma. Another key issue for our consideration is the quality of Chinese scientific publications in these areas. The MLP has not only invested heavily in encouraging Chinese scientists to publish more scientific articles in areas considered to be cutting-edge but has also mandated that the quality of Chinese-authored papers be recognized as among the most highly cited papers in leading international indicators such as the Science Citation Index (SCI). Finally, we want to explore the extent to which the combination of highly qualified scientific researchers and well-appointed research facilities has attracted leading foreign high-tech companies to invest in drug discovery operations in China, an indicator that China is moving up the value chain in leading areas of applied bioscience.

## 2.1 Methods 

Our first goal in analyzing the Chinese nanotechnology scientific research literature is the development of a sort of conceptual map of the evolving Chinese nanotechnology document set. We apply text-mining tools to measure changes in the usage of key terms in Chinese nanotechnology. Specifically, we measure and map the terms included in titles, abstracts, and descriptive labels attached to database records of all papers in Chinese nanotechnology. By measuring the movement of terms much in the way, for example, currency might flow through capital markets we should be able to capture general patterns of growth of terms and concepts in Chinese nanotechnology scientific literature. Through this approach we expect to be able to see how specific research areas rise and/or fall over time, how often such concepts are invoked, and how many times documents with such labels and terms are cited. We should be able to draw a picture of where China is succeeding in nanotechnology by using a representative sample of the totality of China’s scientific publication production. To this end we constructed a Chinese nanotechnology scientific literature database that includes citation data as well as full text abstracts. We then measured changes in frequencies over time in the sample of ISI-assigned topic tags, known as Keywords Plus®. We have used data visualization techniques such as tree maps, bubble maps, geocoded maps and document clusters to assist us in identifying related document sets along with their meanings in the greater document set context. These methods have offered us a robust and strongly inferential means to develop an historical sense of the structure and dynamics of bursts of research interest within isolated conceptual spaces and begin to identify signals of future dominant nanotechnology research areas in China. 

We have analyzed a comprehensive set of metadata on Chinese nanotechnology research articles in order to accomplish several goals. Our first goal has been to measure overall trends in Chinese nanotechnology research production. A second goal has been to identify recent globally significant innovation trends at the intersection of pharmacogenomics and nanotechnology taking place in China. We have focused our data measures on elucidating the impact of the Chinese Guidelines on National Medium- and Long-term Program for Science and Technology Development (2006-2020) on Chinese nanotechnology production. 

The ISI Web of Science (WoS) database contains approximately 40 million scholarly research articles from around the world entirely represented by metadata in English. Each metadata record contains over thirty fields of data including full abstracts. Kostoff et al. specifies a comprehensive query designed to identify nanotechnology documents with a high degree of both precision and recall.^48^ We revised the query in order to specify nanotechnology research articles originating from China stored in the ISI WoS database. Specifically, the query from Kostoff was altered to fit the syntax requirements for querying ISI WoS, adding one clause to specify publications originating from the People’s Republic of China. We executed our revised query in February 2008. We sorted the resulting records by year within the ISI interface and then by the number of times each article is cited (times cited, or TC). Next we selected, for each year, the top 10% of documents according to highest TC. For most years, there was no way to take exactly 10%, so the number of citations C for the top 10% mark was first detected for a given year, and then all articles with at least C+1 citations were selected. (While generating a data set in the way we have is in no way a random sample, ours is a sample deliberately chosen to capture as much of the citation graph of the entire corpus with a small but representative document set. We would normally opt to use the entire corpus but, given the power law distribution of citations, we would benefit most by focusing on the bulk of citations rather than the bulk of articles. By our estimates, the top 10% most cited articles for each year cover approximately 50% of total citation volume for that year.) 

After selecting these records in the ISI WoS interface for download we downloaded the full records containing all ISI WoS metadata fields. The full records were downloaded in groups of 500 articles encoded in plain text files to an Apple MacBook Pro running OS X 10.5. The plain text files containing the records were merged, reformatted and uploaded into MySQL using SQL*Loader into a single master table containing one field for every metadata field in the original ISI set. Further data preparation, such as normalization of individual KeyWords Plus® terms to individual document identifiers, was performed either by creating new tables within MySQL via SQL and PL/SQL to generate and populate the new tables, or by first exporting the data from MySQL, processing the data using Java and/or sed, and then loading the new data set into a new table created using MySQL. Results of queries designed to analyze frequencies and changes of frequencies over time were output to Microsoft Excel and graphed therein.

We measured the change in relative frequencies of ISI KeyWords Plus® over two time frames in order to capture changes over the entire time period. ISI KeyWords Plus® are the most frequently reoccurring terms among the titles of articles the article cites. KeyWords Plus® terms are terms that may not necessarily appear in the article’s own title, abstract, or author-supplied keywords. KeyWords Plus®, according to creator and ISI founder Eugene Garfield, succinctly capture an article’s major and minor themes that may escape close readings of an article, semantic analysis, formal ontology labels, and author keywords.^49^ ISI KeyWords Plus® provide topic labels derived from an article’s local outbound citation network and “act as a surrogate ‘abstract’.”^50^ From here forward, we refer to KeyWords Plus® terms alternately as “topics,” “topic terms,” “topic IDs,” “ID tags,” or “topic tags.” (“ID” is the ISI-assigned field label for the KeyWords Plus® data field.) Another way of looking at it is that we use KeyWords Plus® as the sole feature of a document representation model for modeling topic shifts over time. We chose KeyWords Plus in the hopes it would help us derive an easily-readable representation of the entire document set. By easily readable, we mean a representation that captures some sense of what a collection of documents is “about” that is not limited to the pre-defined boundaries imposed by a formal ontology.

For the preparation of changes in KeyWords Plus® over time, we extracted each individual KeyWords Plus® term occurrence from the master table along with the ISI document identifier associated with that occurrence as well as its year of occurrence. The list of KeyWords Plus was grouped into term-by-year, and the frequency of use of each term for every year was counted. The total instances of all KeyWords Plus® terms used per year were counted as well. Finally, the percentage of the total term space for each year was calculated for each term, and an average for all terms for each year was derived. Visualizations of KeyWords Plus® terms were generated using IBM’s ManyEyes data visualization web service.

The two time frames we chose were 2004-2005 (1304 articles in the database) and 2006-2007 (1692 articles in the database). We chose the end of 2005/start of 2006 as a temporal breakpoint because it corresponds to the introduction and implementation of the MLP at the start of 2006 and its first potential impacts on Chinese nanotechnology production. 

We chose to detect substantive change over a set of documents by measuring relative frequencies of KeyWords Plus® terms over time as a way of modeling topical shifts in a dynamic and evolving corpus. Our approach to measuring topical shift in Chinese nanotechnology after the implementation of the MLP involves measuring and comparing changes in what we call the “factor above average” (FAA) for every KeyWords Plus® term in the document set for both time periods. FAA is simply the factor by which a topic term (or, in this case, a KeyWords Plus® term) exceeds the average topic term’s frequency during a given time period. Given these “factor above average” (FAA) measures for all topic terms for both time periods, we then compared change in factors for leading topic terms from 2004-2005 to 2006-2007, giving to us the specific topics that had grown most radically as attention-getters. We detected the largest gainers by measuring the change in a topic term’s FAA from the two years before MLP implementation to the subsequent two-year period using the following equation, where Ti is the ith topic term T: 


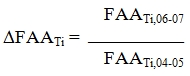


For example, if the KeyWords Plus® term ‘CARBON NANOTUBE’ was used 28 times during 2004-05, and 789 other merged KeyWords Plus® terms were used a total of 2095 times during 2004-05, we know there are 790 unique KeyWords Plus® terms used a total of 2123 times, meaning a KeyWords Plus® term was used an average of 2123/790, or 2.687 times, during 2004-2005. To calculate the Factor Above Average for the topic term ‘CARBON NANOTUBES’ during 2004-05, we would simply divide the number of times ‘CARBON NANOTUBES’ was used during the period by the average times any term was used. In this case, the FAA2004-05 of ‘CARBON NANOTUBES’ is 28/2.687, or 1042%. If the FAA2006-07 of ‘CARBON NANOTUBES’ is 1585%, then the ΔFAA of ‘CARBON NANOTUBES’ is 1585/1042, or 1.52, or 152%.

By ranking the full list of KeyWords Plus® terms ranked by FAATi we are able to identify the leading topic terms and use them as indicators of types of rapidly growing practices, discourses, and production. Using these statistics we can generate visualizations of the conceptual landscape within Chinese nanotechnology and identify conceptual trends, controlling for growth of the overall space for each topic’s respective time period. Obtaining these terms will help us refine our process of identifying areas of growth within Chinese nanotechnology. Specifically, once we obtain a list of top KeyWords Plus® terms we may use these terms not only to identify rising subject areas but also to formulate a high-precision query for the research collection, leading to more specific data and more detailed visualizations. The set of all rising terms may also indicate more broad trends across the entirety of Chinese state production and identify trends stimulated or created by the implementation of the MLP.

Just as we created a database of all Chinese nanotechnology publication records from ISI WoS, we also created a similar collection for all US nanotechnology publications. This data collection was used primarily in the present study to evaluate claims about the quality of Chinese nanotechnology publications by looking at the impact of Chinese collaborators on US-based publications while comparing that impact to all collaborators and international collaborators. If we merely count citation frequency averages for each year in the Chinese nanotechnology research corpus and compare those averages to those of the US, we will not be able to control for culturally specific biases regarding citation behaviors. If we were to compare China to US directly, we would not know whether the lower citation frequencies in the Chinese literature is lower because the Chinese literature is of lower interest and quality or because members of the Chinese research community simply cite less frequently than their US counterparts. If the impact of a Chinese collaborator does not affect the times cited measure (TC) of a paper when compared to all collaborative papers, then Chinese quality cannot fairly be considered inferior. Further, if this comparison is put in light of a comparison between the frequency of US citing behaviors and Chinese citing behaviors, with Chinese citing behaviors being lower, then it remains difficult to support the claim that Chinese nanotechnology is of inferior quality.

##  3.1 Results

**Figure 1:**
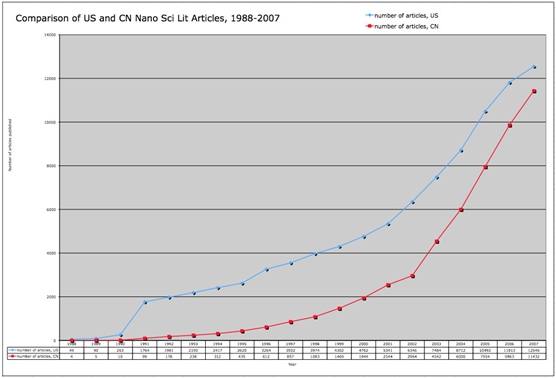
Comparison of total nanotechnology articles published per year in the US and China, 1988-2007 (source: ISI Web of Science, Feb 2008)

**Figure 2:**
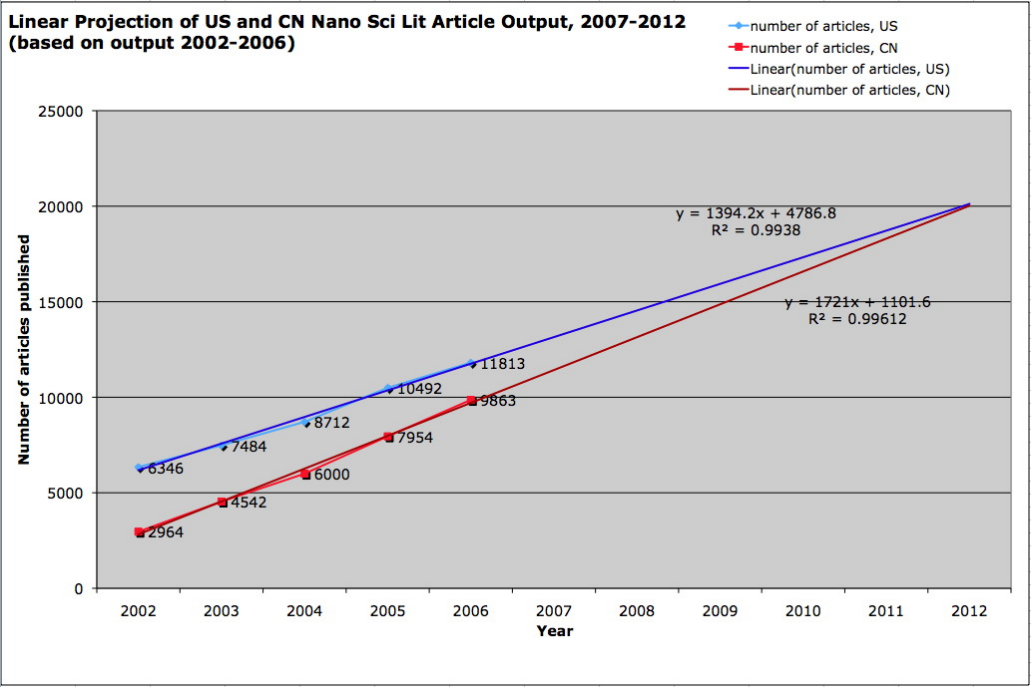
Linear projection of US and Chinese nanotech literature output per year, 2007-2012 (source: ISI Web of Science, Feb 2008)

**Figure 3:**
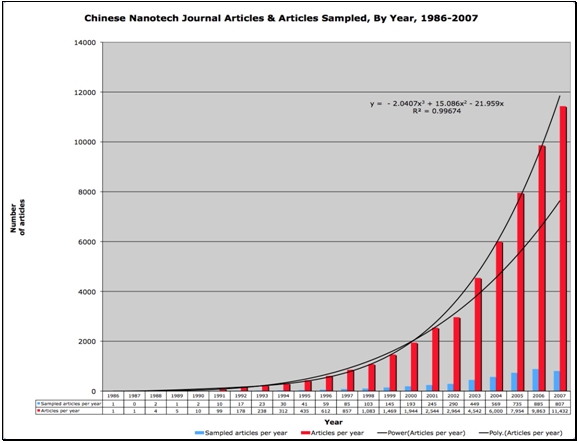


As noted in section 1.2 above, China is enjoying rapidly growing investments from the public sector and even more rapid increases in investments from private sources, helping to transition China into a knowledge economy at a rate possibly unmatched by any other nation. China rose to become the number two ranked nation in the world in terms of total number of scientific publications produced as of 2007.   According to Zhou and Leydesdorff, China’s scientific publication production is growing at an exponential rate, as is the frequency by which its papers are cited by the global scientific community.   Further, Zhou and Leydesdorff argue that China is second only to the United States in nanoscience and nanotechnology production. Our data support these arguments; figures 1 and 3 show that China is increasing its production of nanotechnology publications at an exponential rate. Figure 2 provides linear projections of Chinese and US publication production based on productivity from 2002-2006. Based on the rates of change implied by these projections the nanotechnology productivity volumes of the US and China appear to cross sometime around 2012. 

As we have noted above, among the major objectives of the MLP is to enhance the quality of its research products in terms of TC. A June 2008 editorial in Nature Nanotechnology agrees that China will soon overtake the US in terms of nanotechnology paper production.  However, the editorial insists that the US will continue to lead the world in terms of quality of its research, thus apparently mitigating any concern about the leadership of US nanotechnology production and its competitiveness in comparison to other nations. 

The inferiority of China’s scientific capacity seems to support claims that Chinese nanotechnology research quality is inferior. In her recent book, The New Invisible College: Science for Development Caroline S. Wagner argues that China ranked 47th in the world in total scientific capacity as of 2000.  

  In our view the evaluations of the quality of Chinese nanoscience by the June 2008 Nature Nanotechnology editorial, including an inference from low scientific capacity that quality may be low, may be missing the mark. While China may have ranked relatively low in terms of scientific capacity prior to 2000 enormous progress has been made since that time in the development of the infrastructure supporting science and technology as we have shown above. As we will show in more detail below, in some specific areas of research such as bionanopharma the progress is quite extraordinary.  Moreover, we feel that the methods used in the Nature editorial do not provide a sufficient picture. Crucially, the Nature editorial measures quality by the number of times an article is cited, a commonly accepted quantitative indicator of the quality of a paper in the field of scientometrics. Despite the measure’s common acceptance, significant questions arise regarding the use of this measure to compare the quality of scientific output from two nations.  Is this editorial’s estimate of China’s inferior quality accurate or fair?   Preliminary indications seem to support the claim that Chinese research quality is inferior; after all, Chinese nanotechnology is inferior to US nanotechnology in terms of how many times each nation’s nanotech research corpus is cited. We have found that while the 10 most impactful US nanotechnology papers number among the 50 most cited nanotech papers in China, not one of the 50 most impactful Chinese nanotech papers number in the US’s top 50 most impactful nanotech papers.  The best US papers are cited far more often than the most cited Chinese papers.  

 It seems, however, that the editorial’s use of the measure of quality applied to a comparison of the output of the two nations may be flawed in at least three ways. (1) Does the lower times cited (TC) volume and TC per paper average for Chinese papers indicate inferior quality, as the editorial suggests, or does that measure actually obscure a passing phase of inferior quality for China? Another way to ask this question is to ask, are the differences in TC statistics for Chinese and US decreasing? (2) Further, when comparing between two nations, might such a citation count obscure a basic cultural difference in attitudes and use of citations between the two nations? Could it be that Chinese scientists simply cite fewer papers on average? (3) Finally, can we make such claims given that there is a significant degree of collaboration between scientists in the two nations?  

Statistics generated from an analysis of our nanotech corpora indicate that the answer to the second question is that the average number of outbound citations per US nanotechnology paper has been stable at approximately 34. While the number of outbound citations by Chinese nanotech papers was below that of the US as of 2005, it has been on the rise, having risen from 24 to 27 per paper from 2001 to 2005 (versus 34 at 2001 and 2005 for the US collection). Clearly any direct measure of citation volume as a proxy for quality will be significantly biased against China.  The answer to question (3) holds the key to answering question (1). That is, we might be able to test the inferior quality hypothesis by comparing apples to apples in one nation’s context.  Instead of comparing Chinese articles to US articles, we may be able to focus solely on US papers.  As stated above in the methods section, by comparing how US and Chinese papers are cited by US papers, we can control for the cultural difference in citation behavior and control for the impact of collaboration. If Chinese nanotechnology research is of inferior quality we should expect that Chinese nanotech research is cited less often than US nanotechnology research in the same context, namely, in the same corpus.  Specifically, we should see that Chinese collaborators on US papers should bring down TC rates over the average of collaborative papers in the US. 

As illustrated by Table 1 the “Chinese effect” on citation volumes in US nanotech literature is significantly positive, in contrast to all publications, collaborative publications, and even in contrast to all international collaborations. This finding makes it difficult if not impossible to support the notion that Chinese nanotech research is of an inferior quality. Chinese-US collaboration improves impact of US research and Chinese research alike in the US nanotech corpus.  As can be seen in Table 1, collaborative papers are cited about as often as all publications and, after 2003, about as often as all papers with international collaborations. US-Chinese collaborations are cited approximately 30% more often than all publications in the US nanotech corpus over 1998-2007, approximately 26% more often than other collaborations.  In only two of the ten years (1998 and 2007) did the presence of Chinese collaborators have a negative impact on the number of times cited.  When US papers in the sample feature international collaborators, they tend not to add any citation benefit. Yet when Chinese collaborators are added, the average impact is significantly positive. Further, the positive impact of collaborations with Chinese collaborators appears to be on the rise.

**Table 1:**
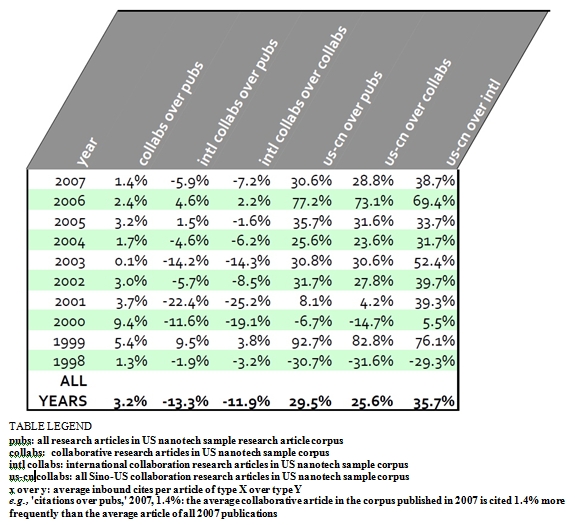


Figure 3 below shows the number of Chinese nanotech research articles produced per year as well as our data sample size.  As is shown in Figure 3, the total set of Chinese nanotechnology research publications equals 52,909 articles, 35,249 of which were published from 2004-2007.  Our sample contains 2996 relevant articles from the period 2004-2007, roughly 8.5% of the 35,249 Chinese nanotech articles.

**Figure 4:**
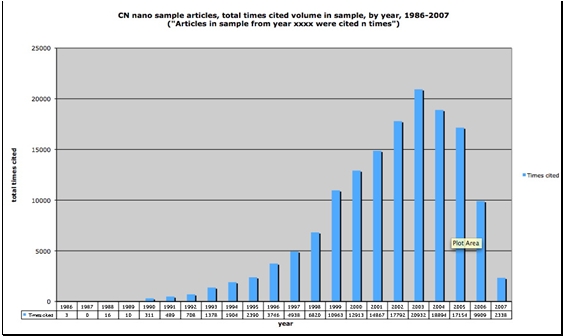
Volume of citations per year in document sample set, 1986-2007, Chinese nanotechnology documents  (source: ISI Web of Science, Feb 2008)

**Figure 5:**
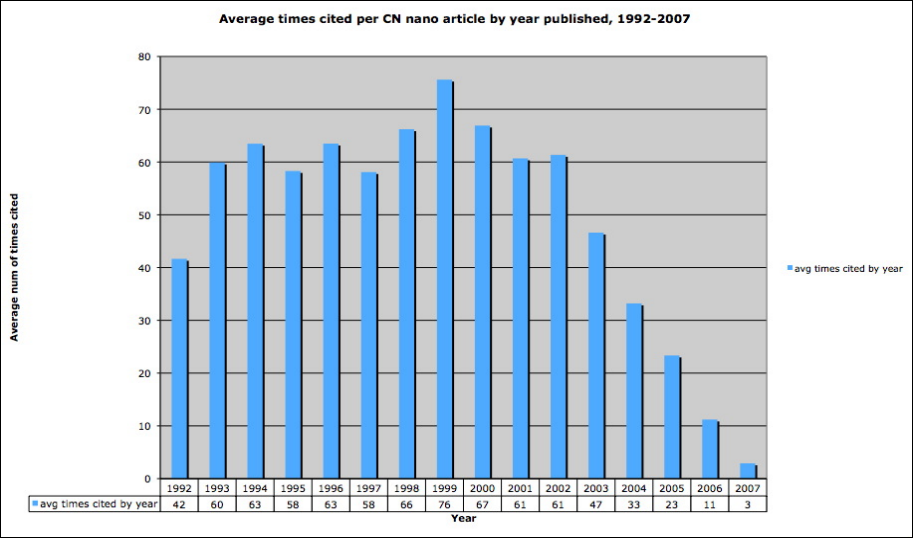
Average number of citations per article, by year published, 1992-2007, Chinese nanotechnology (source: ISI Web of Science, Feb 2008)

Figures 4 and 5 below illustrate changing citation behavior in Chinese nanotechnology. While the number of articles is steadily increasing as shown in Figure 3, the citation volumes and number of citations per year from 1999-2004 as depicted in Figures 4 and 5 are decreasing. The decrease occurs not because papers from these years are decreasingly impactful but rather because citations as a measure provide us with a time-dependent measure, particularly a lagging one. The data shows that citation-dependent measures trail measures of internal document features by approximately four to eight years. In other words, our most current findings about Chinese nanotechnology based on citation data can be no newer than four years old. In order to circumvent this lag effect inherent in citation measures, we propose that measuring internal features of documents such as terms including KeyWords Plus® may give us more current information than citation-dependent measures. KeyWords Plus® provide the sort of internal document features that we can use to build a sensitive measure of shifting trends in the nanotech literature of the very recent past. With document dependent measures we won’t have to wait years before detecting important trends in Chinese nanotechnology. 

**Figure 6:**
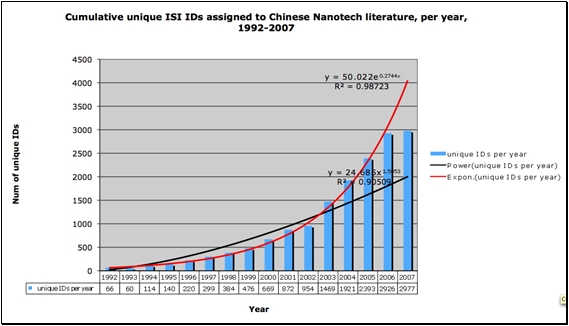
Cumulative unique ISI-assigned KeyWords Plus® terms, Chinese Nanotech research literature, per year, 1992-2007. (source: ISI Web of Science, Feb 2008)

**Figure 7:**
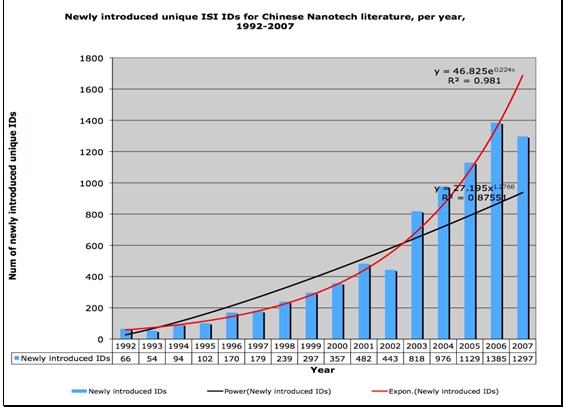
Newly introduced unique ISI KeyWords Plus® terms for Chinese Nanotech research literature, per year, 1992-2007  (source: ISI Web of Science, Feb 2008)

In an important sense, the creation of new KeyWords Plus® terms is indicative of the growth of new ideas. Also, measuring the proliferation of topic terms gives us a direct measure of changes in the diversity of topics. Figures 6 and 7 illustrate the proliferation dynamics of topic terms in Chinese nanotechnology over time. 

We can see from Figures 6 and 7 that topic term dynamics indicate that Chinese nanotechnology is a growing field both in terms of total topic terms as well as newly introduced topic terms. What is also visible in Figures 6 and 7 is that, unlike citation-based measures, topic term-based measures do not lag behind current growth patterns by several years. KeyWords Plus®-based measures give us very current measures, measures that are distributing exponentially just as the research paper set data measures are. While we expect the total volume and proliferation of different topic terms to go up as the number of papers increases, we should not take for granted that new ones will increase with the same magnitude as the increase in the number of publications. 

**Figure 8:**
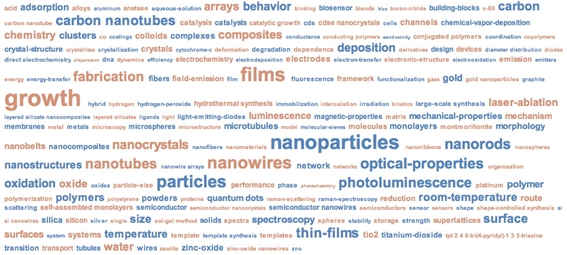
CN nanotech-relevant KeyWords Plus® terms  multiplied by times cited, raw KeyWords Plus® terms data, 1992-2007. (source: ISI Web of Science, Feb 2008)

Figure 8 is a visualization of the most frequently occurring KeyWords Plus® terms over the entire document set multiplied by the times documents associated with those topic terms are cited. Figure 8 can be thought of as representing the relative visibility of specific topics in Chinese nanotechnology since 1992. From it we can see the relative importance of such topics as nanoparticles, photoluminescence, films, nanotubes, growth, and nanowires, among others. Note that this visualization is a static measure of the entire historical frame of Chinese nanotechnology and does not capture the dynamics of Chinese scientific production in the field. 

Our analysis relies upon adequate coverage of KeyWords Plus® terms for our data set.  Table 2 below shows KeyWords Plus® are used for nearly 100% of the documents in the sample data set.

**Table 2:**
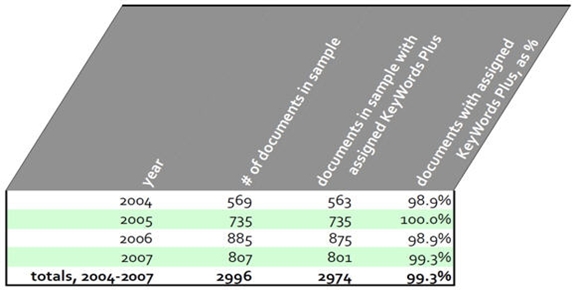
Percentage of documents in the sample data set represented by KeyWords Plus® terms

Table 33 below shows a ranked list of the KeyWords Plus® terms showing the greatest increases in Chinese nanotechnology from 2004-05 and from 2006-07.

**Table 3:**
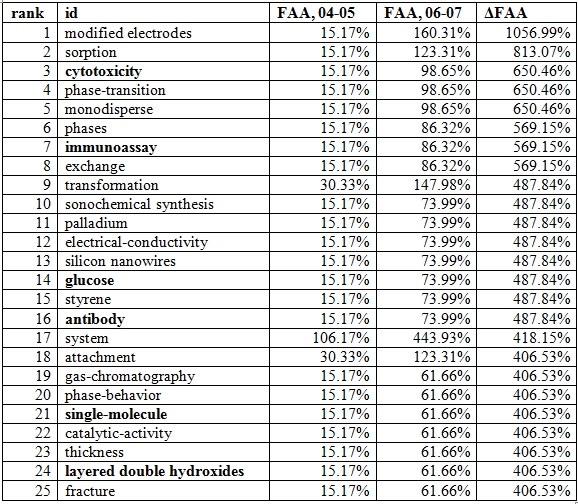
KeyWords Plus® terms showing largest increase in frequency, Chinese nanotech research lit, 2004-05 to 2006-07

Particularly noteworthy for our analysis is that six of the 25 topic terms (in Table 3, in bold) are either exclusively (cytotoxicity, immunoassay, glucose, antibody) or primarily (single-molecule, layered double hydroxides) related to bionanotechnology and pharmaceutical nanotechnology. Of the 1006 topic terms that occurred in both time frames, only 460 showed increases in usage. Of those 460, it was found that a large number of them are important to bio- and pharma- nanotechnology—not just the ones in bold in Table 3. Table 4 shows the remaining KeyWords Plus® terms (those not already in table 3) showing growth above 200% that are almost always deeply relevant to bio- and pharma- nanotech. 

**Table 4:**
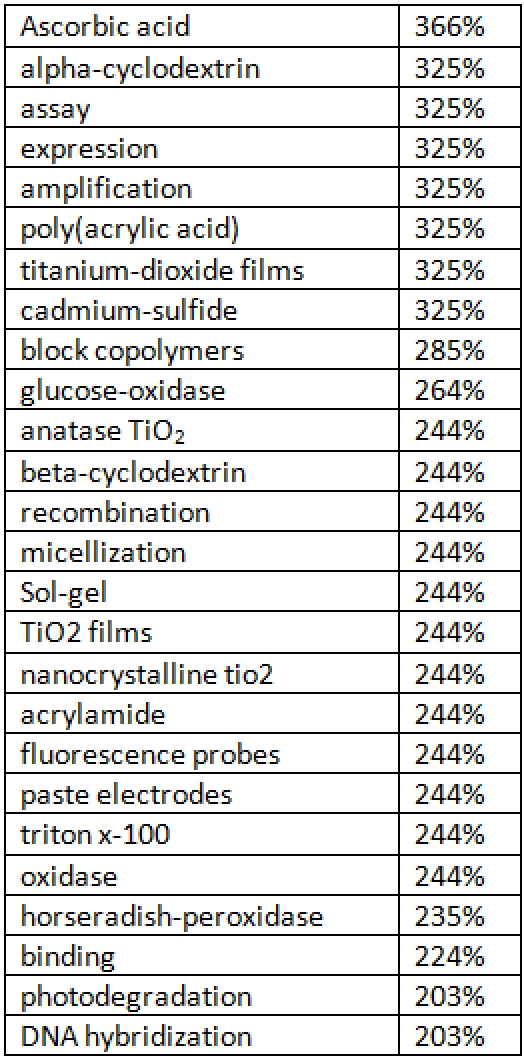
KeyWords Plus® terms showing largest increase in frequency, Chinese nanotech research lit, 2004-05 to 2006-07

## 

In total, out of the 160 topic terms that have grown in usage by at least 200%, 32 (20%) are at least strongly if not exclusively relevant to bio- and pharma-nanotechnology. Given that some of the other fastest-growing topics may be relevant or are at least not irrelevant to bio- and pharma- nanotechnology, we may conclude that a large amount of topical growth in Chinese nanotechnology from 2004-05 to 2006-07 is specifically related to bio- and pharma- nanotechnology. 

Next, we used the set of 32 bio- and nanotech- relevant nanotech KeyWords Plus® terms shown in Tables 3 and 4 above in order to formulate a high-precision^56^ query for bio- and pharma- nanotechnology documents. The query, written in SQL, searched for all records whose KeyWords Plus® field matched any of the 32 terms from tables 3 and 4.

mysql> select * from cn_nano_IDs_0607 where ID like ’ cytotoxicity’ or ID like ‘immunoassay’ or ID like ‘glucose’ or ID like ‘antibody’ or ID like ‘single-molecule’ or ID like ‘layered double hydroxides’ or ID like ‘Ascorbic acid' or ID like 'alpha-cyclodextrin' or ID like 'assay' or ID like 'expression' or ID like 'amplification' or ID like 'poly(acrylic acid) ' or ID like 'titanium-dioxide films ' or ID like 'cadmium-sulfide' or ID like 'block copolymers' or ID like 'glucose-oxidase' or ID like 'anatase TiO2' or ID like 'beta-cyclodextrin' or ID like 'recombination' or ID like 'micellization' or ID like 'Sol-gel' or ID like 'TiO2 films' or ID like 'nanocrystalline tio2' or ID like 'acrylamide' or ID like 'fluorescence probes' or ID like 'paste electrodes' or ID like 'triton x-100' or ID like 'oxidase' or ID like 'horseradish-peroxidase' or ID like 'binding' or ID like 'photodegradation' or ID like 'DNA hybridization';

We used the query on the 1692 Chinese nanotechnology documents in our sample set that were published during 2006-07 (as in the code example above) as well as the 1304 documents in our sample from 2004-05. Fully 209 (12.4%) of the 1688 papers from 2006-07 Chinese nanotech are identified by these topic terms, up from 8.6% (112 of 1304) during 2004-2005. In other words, approximately one-third of the increase of Chinese nanotech research publication production from 2004-05 to 2006-07 is identified by these 32 bio- and pharma-relevant topic terms alone. On the one hand we know that the 32 topic terms used to formulate the query do not fully cover the Chinese bionano space and on the other we know that the documents represented by these 32 terms are not necessarily all bio- or pharma- nanotechnology-related. For example, papers labeled “block copolymer” may not all be bionano papers. As another example, we might have a drug nanosensor paper that does not have any of the 32 bionano-indicating topic terms assigned to it. 

**Figure 9:**
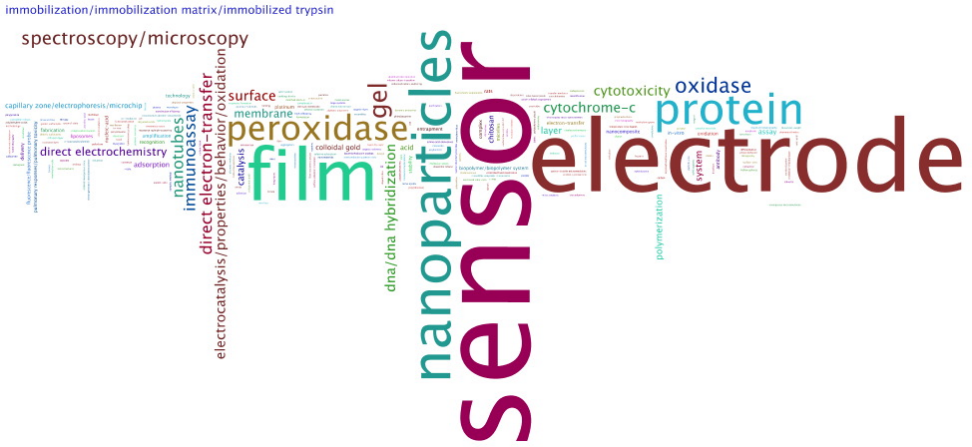
Wordle, KeyWords Plus® term frequencies, CN bionano research, 2006-07. (source: ISI Web of Science, Feb 2008; graphics generated using IBM ManyEyes)

Because of this ambiguity, we took a close look at all 209 documents generated from the query from the 32 terms, first by visualizing the topic term space and then by reading each document in the result set. In other words, if the above KeyWords Plus® terms are used as a query, records will be generated that contain a larger set of KeyWords Plus® terms. From that larger set we created three KeyWords Plus® term frequency visualizations of the 209 bionano-relevant papers from 2006-7, shown in Figures 9 through 11 below. Each visualization serves differently to both confirm the high relevance of these documents to bionano and illustrate the relative importance of different topical areas within the subdomain: Differently, because each of the visualizations captures the same measure but displays the data differently. 

**Figure 10:**
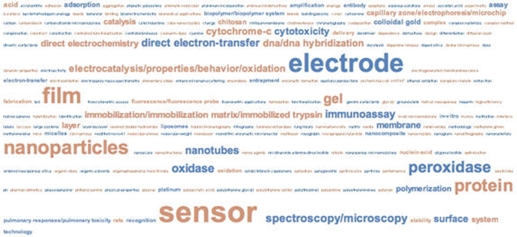
Tag cloud, KeyWords Plus® term frequencies, CN bionano research literature, 2006-07. (source: ISI Web of Science, Feb 2008 ; graphics generated using IBM ManyEyes)

**Figure 11:**
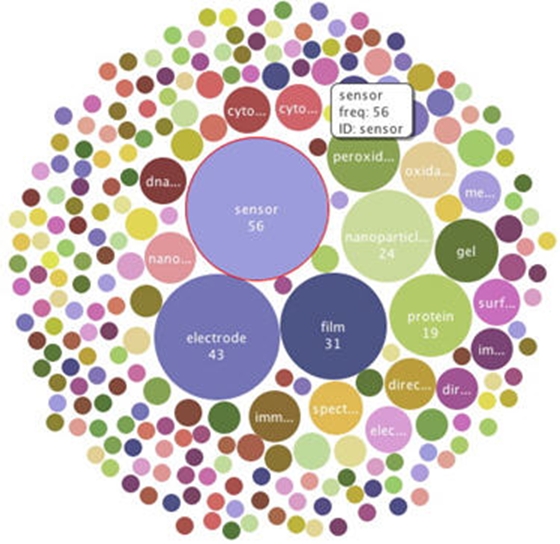
Bubble chart, KeyWords Plus® term frequencies, rapid growth areas in CN bionano research lit, 2006-07. (source: ISI Web of Science, Feb 2008; graphics generated using IBM ManyEyes)

Next we took a closer look at the documents retrieved by the queries, reading the 209 papers to examine the papers for their relevance to pharmaceutical bionanotechnology. We discovered that at least 115 of the 209 papers initially deemed relevant to bionano were unambiguously and strictly relevant to pharmaceutical bionanotechnology. These 115 papers comprise 6.8% of all Chinese nanotechnology publications for the 2006-2007 period. They also represent 911 of 12,247 (7.4%) inbound citations for the period. A reading of those 115 documents shows that they are specifically focused on topics related but not limited to biosensors, immune sensing, glucose sensing, disease detection, DNA analysis, drug release, metabolics, and neurosurgery. 

## 3.2 Discussion

As with Chinese scientific production and nanotechnology, the biopharmaceutical industry in China has demonstrated explosive growth in recent years. It may be no surprise, then, that our results demonstrate that biopharmaceutical nanotechnology is one of the fastest-growing areas within nanotechnology in China.

Over the five years leading up to China’s implementation of the MLP, issued in December 2005, the Chinese pharmaceutical industry tripled in size to US$3B^57^, a market estimated as of late 2008 to be in the neighborhood of US$10B. Recently, GlaxoSmithKline (GSK), Roche, Sanofi-Aventis, and Novo Nordisk have established R&D centers within China. For example, Sanofi-Aventis’ expansion includes a Clinical Research Unit attached to the state-of-the-art Biometrics facility in Beijing and a partnership with Shanghai Institutes for Biological Sciences (SIBS) for drug discovery, motivated according to Sanofi and SIBS officials by the desire to tap into the Chinese R&D talent pool.^58^ In addition, Sanofi-Aventis set up an organization in China to boost its network of collaborations with small biotech companies, public laboratories, and research institutes.^59^ Sanofi-Aventis also created a scholarship program to recognize and develop the most promising Chinese scientists engaged in cutting-edge pharmaceutical R&D in the fields of structural chemistry, biology and pharmacology.^60^ Additionally, In April 2008, Genzyme announced intentions to construct a new R&D center in Beijing’s Zhongguancun Life Science Park to establish a long-term presence in China. The new US$90 million facility is expected to open in 2010 and employ 350 people conducting R&D on orthopedics, transplantation, immune disease, oncology, endocrinology and cardiovascular disease. 

GSK’s strategy in China is typical of the approach being followed by other leading pharma companies. At its formation in 2000 GSK broke with the traditional model of organizing research in a single large-scale facility and established instead “Centers of Excellence in Drug Discovery” (CEDDs) consisting of 11 different integrated groups of scientists and clinicians located in the US, UK, and Europe organizing their work around specific disease areas, with the intent to produce nimble and entrepreneurial discovery units.^61^ In 2005 this model was expanded to take advantage of pockets of research excellence developing in other parts of the world, such as China and India. These new Centers of Excellence for External Drug Discovery (CEEDDs) have the same goals as the CEDDs of delivering medicines into late-stage development, but do so by establishing and managing long-term strategic collaborations with biotech and small-to-medium-sized pharmaceutical companies. As part of this strategic intent, in 2007 GSK established a dedicated R&D center in Shanghai, focused on research into neurodegeneration with the objective of creating new medicines for such severe disorders as multiple sclerosis, Parkinson’s disease and Alzheimer’s disease. As of 2008 the Shanghai R&D center had recruited 200 scientists, including experienced researchers dedicated solely to GSK’s neurodegenerative research.^62^ The center will eventually direct the global discovery and development activities within its therapeutic area, from drug-target identification to late-stage clinical studies, while collaborating with research institutions elsewhere in China and other countries. 

In addition to Western pharmaceutical giants relocating R&D efforts within China, such companies are also increasingly dependent on contract research organizations (CROs) to carry out much of the research previously performed in house within such multinational pharmaceuticals. A CRO is any organization that provides any of a number of outsourced research services. CROs are diverse and offer services ranging from product development, manufacturing, biostatistical analysis, clinical trial sample testing, and in some cases, full clinical trial management through all phases of the regulatory pipeline. The global CRO market is predicted to reach at least US$36 billion by 2010.^63^ The story of CROs in China shares a theme with pharma multinational R&D expansion in China: talent.^64^ A number of CROs such as WuXi PharmaTech were founded by Chinese researchers returning from graduate study and employment abroad.^65^ Chinese graduate students, traveling abroad to receive their advanced degrees, are increasingly returning home due to emotional and financial incentives, bringing home the ability to run advanced scientific operations,^66^ fueling the value and growth of Chinese CRO operations, and, in turn, further incentivizing relocation of large vertically-integrated R&D operations to China. According to China CRO 2009, an industry compilation assembled by Modular R&D of all global and local CROs operating in China as of April 30th, 2009, 122 CROs in drug discovery, preclinical studies and clinical trials are currently operating in China. According to a recent report from PricewaterhouseCoopers, the CRO market in China was US$186 million in 2007. That number represented growth of 38% from the previous year, an increase that was mainly driven by outsourcing from international firms rather than domestic biopharma companies. In the future it is estimated that the total China CRO market will grow to US$791 million in 2012, comprising 2.3% of the global CRO market. 

Another driver for moving R&D overseas is the loss of economic value of drug discovery in the West as a business model, often referred to as the failure of the “blockbuster model.” Discovering a new drug is so costly that it is nearly impossible for a company to reach a break-even point, creating incentive for moving the labor of lead discovery and optimization to China.^67^,^68^While lowered costs in combination with talent and the availability of CRO services is an important driver for moving pharma R&D to China, it does not account for all of the motivation. It is also important that China has manufactured pharmaceutical precursors for years, and it is presently increasing production in this area. China has a very large population with a rising middle class, meaning more customers. 

While China’s investment in biotechnology is low in comparison to countries such as the US, as we have noted above, China has become the world’s second-leading investor in nanotechnology and is now the second-leading producer of nanotechnology research articles and on the way to becoming the leader by 2012. China’s system of innovation, benefitting from systemic optimalities, has gone far in supporting China’s leap-frogging efforts in nanotechnology. It is not surprising, then, given the interest of foreign investment in pharmaceutical drug discovery and research in China along with the Chinese push to grow basic nanosciences, that a large proportion of nanotechnology is focused at the intersection of nanotechnology and drug discovery, a combination that may be attractive to drug companies in a post-blockbuster era looking to explore bio-based discovery, pharmacogenomics, and nanoscale high throughput clinical data collection methods.

## 4.1 Conclusions  

Since the introduction of the new Chinese S&T policy guidelines in 2006, a significant component of rapidly rising Chinese nanotechnology is led by overt medical, drug, and genomic applications. These papers increasingly focus on areas of research crucial to neurosurgery, biosensors (specifically immune sensing, glucose sensing, disease detection, DNA analysis), drug release, and metabolics. The 50% increase in Chinese bionano and nanopharma research in the wake of the MLP generated at the barest minimum one third of the growth of Chinese nanotechnology research resulting from the plan. While this finding alone seems surprising, it corresponds to reports of changes in the global pharmaceutical industry.  As drug discovery becomes increasingly dependent upon biosensor technology, and as large pharmaceutical corporations increasingly shutter their US- and UK-based internal discovery programs while shifting investments to external organizations for discovery, China has undertaken heavy investment in innovative bionano corporations to meet global pharmaceutical demand.  When we consider, for example, recent layoffs in the US and UK by GlaxoSmithKline  with corresponding recreation of those positions in China  alongside the proliferation of financial moves such as China Medical’s acquisition of an HPV-DNA biosensor system, or the recent creation of the SIP Bio & Nano Technology Development Corporation, we can begin to understand the forces driving the sudden upsurge in applications-driven bionano research in China. 

 The assessment of the claims made in the present paper will require qualitative analysis, field work and interviews of participants in various segments of Chinese bionano and pharma industry. Such qualitative analysis should serve not only to validate the specific results but also to evaluate the methods we have used here.   To that end, preliminary field work performed by one of the authors in August 2009 in Beijing, Shanghai, Suzhou and Hangzhou has confirmed a massive focused effort by China to support entrepreneurial activity and research facility development pairing biotechnology and nanotechnology. One striking feature of this effort discovered in our field work is the creation and development of China’s two largest nanotechnology research institutes, institutes that will open their doors at the head of a newly-built massive pharmaceutical industrialization park in Suzhou, a project directly created by implementation of the MLP.  Most revealingly, the nanotechnology research centers are being developed in close coordination with the neighboring bionano incubation facilities.

A close reading of the entirety of nanotechnology research production is necessary if we are to understand the landscape of all specific forms of nanotechnology research currently expanding in China, an expansion that cannot be measured sufficiently through any combination of dollars, citations or actors. An adequate study requires we measure the output of terms. The measurement of topic frequencies, specifically KeyWords Plus® frequencies, associated with research documents over time gives us a finely grained image of the specific areas of growing areas of interest in Chinese nanotechnology that we cannot obtain with sufficient detail by restricting ourselves only to more qualitative and better-established means. An analysis of topic tags across a large corpus can, in the words of ISI creator Eugene Garfield, “act as a surrogate ‘abstract’” for the entire corpus, a dynamic abstract that reveals trends of differing scales therein.
